# Patient-Specific, Multi-Scale Modeling of Neointimal Hyperplasia in Vein Grafts

**DOI:** 10.3389/fphys.2017.00226

**Published:** 2017-04-18

**Authors:** Francesca Donadoni, Cesar Pichardo-Almarza, Matthew Bartlett, Alan Dardik, Shervanthi Homer-Vanniasinkam, Vanessa Díaz-Zuccarini

**Affiliations:** ^1^Mechanical Engineering, University College LondonLondon, UK; ^2^The Department of Surgery, Yale University School of MedicineNew Haven, CT, USA; ^3^Veteran Affairs Connecticut Healthcare SystemWest Haven, CT, USA; ^4^Leeds Vascular Institute, Leeds General InfirmaryLeeds, UK; ^5^Division of Surgery, University of WarwickWarwick, UK

**Keywords:** neointimal hyperplasia, vein grafts, remodeling, shear stress, computational fluid dynamics, multi-scale modeling

## Abstract

Neointimal hyperplasia is amongst the major causes of failure of bypass grafts. The disease progression varies from patient to patient due to a range of different factors. In this paper, a mathematical model will be used to understand neointimal hyperplasia in individual patients, combining information from biological experiments and patient-specific data to analyze some aspects of the disease, particularly with regard to mechanical stimuli due to shear stresses on the vessel wall. By combining a biochemical model of cell growth and a patient-specific computational fluid dynamics analysis of blood flow in the lumen, remodeling of the blood vessel is studied by means of a novel computational framework. The framework was used to analyze two vein graft bypasses from one patient: a femoro-popliteal and a femoro-distal bypass. The remodeling of the vessel wall and analysis of the flow for each case was then compared to clinical data and discussed as a potential tool for a better understanding of the disease. Simulation results from this first computational approach showed an overall agreement on the locations of hyperplasia in these patients and demonstrated the potential of using new integrative modeling tools to understand disease progression.

## 1. Introduction

Peripheral bypasses are amongst the most common vascular interventions; however, the reality is that millions of these bypasses fail due to vascular remodeling and this is a real burden for National Health Systems. In the UK alone, costs for surgery exceed 200 M (Peach et al., 2012). Why bypasses fail is a critical issue in vascular surgery today, however, traditional approaches have not provided answers to this problem. It is essential to mention that as of today, animal experiments to study peripheral grafts have failed dramatically. To put it simply, there are no animal models which would provide useful data to understand lower extremity venous bypass failure in humans and other, novel approaches are urgently required. The ideal vascular conduit remains the “Holy Grail” of vascular surgery (Byrom et al., 2010). Variability and uncertainty of the outcome are serious issues and the consequences of a failed graft for every patient, just dire. When a bypass graft blocks, blood supply is usually worse than before bypass surgery. In these circumstances amputation can be inevitable unless the graft can be salvaged and the blood supply restored. Recent randomized controlled trials showed that 40% of lower extremity vein grafts occlude or develop significant stenosis within the first year after implantation (Owens et al., 2015). Results for more complex procedures to the calf vessels have usually slightly worse prognosis, with resultant serious morbidity and mortality. These figures have largely remained unchanged for the past several decades. One can read (Owens et al., 2015), “…it is discouraging to consider that 5 decades of high-powered science has not effectively changed bypass graft outcomes.” Improving results of long-term limb salvage remain critically dependent on understanding the mechanisms of successful vein graft adaptation to the arterial environment. In a clinical landscape with ever-increasing and more aggressive bypass procedures, the use of novel mathematical modeling and simulation tools to understand venous adaptation to the arterial environment would help preventing the significant numbers of excess complications, mortality and cost of re-interventions and alternative therapies. This is key in order to devise a personalized “best management plan” and to deliver the best treatment for a specific patient, at that crucial time.

This paper is focused on the development of a simulation framework to elaborate the mathematical tools needed to understand and model key properties of a multi-scale, clinical problem, i.e., bypass failure, at different levels. The vision is that we can use multi-scale mechanistic models to understand and disentangle the complexity of lower limb graft failure and to start making some headway toward patient stratification in this regard.

Neointimal hyperplasia (NIH) is one of the processes leading to restenosis (and ultimately graft failure). NIH is, in simple terms, the re-narrowing of a blood vessel after a stenting or vein grafting surgical procedure, due to tissue growing at the site of injury (Murphy and Boyle, 2010). Upon the start of the formation of NIH, smooth muscle cells change their phenotype from contractile to synthetic, making them more proliferative and resulting in thickening of the arterial tissue. The new conditions in the vascular tissue trigger a reaction which also leads to the release of growth factors and cytokines, including transforming growth factor beta (TGF-β) (Guerri-Guttenberg et al., 2013), platelet derived growth factor (PDGF) (Huang et al., 2002), fibroblast growth factor (FGF-2) (Nabel et al., 1993) and a group of inflammatory cytokines (Collins et al., 2012). Furthermore, other hypotheses have been studied such as that the fibroblasts in the adventitia might move to the media in the form of smooth muscle cells or that bone marrow-derived progenitor cells coming from the bloodstream might also turn into smooth muscle cells to form part of the vascular tissue (Collins et al., 2012). In addition to smooth muscle cells, other types of cells, such as monocytes, are also involved in intimal volume growth (Stark et al., 1997), in response to the inflammatory process triggered by the surgical cut.

However, studies have shown that biological mechanisms alone are not the sole process leading to changes in the morphology and geometry of the artery. A number of mechanical forces in the arterial wall, and shear stress also play a role (Owens, 2010). As opposed to artificial stents, in the case of vein grafts it is the whole conduit, and not just the arterial tissue around it, to be made of living tissue and thus to respond to the surrounding environment. As a result, it is particularly important to consider both the mechanical and the biological mechanisms behind remodeling. Given the complex interplay between biological mechanisms and mechanical stimuli, mathematical modeling can offer much needed help.

Previous research has shown the potential of computational modeling to describe the connection between hemodynamic factors and NIH. One of the first studies to model the relation between blood flow dynamics and NIH appeared in 2001 (Hill and Spendiff, 2001). In this model, the relationship between flow and tissue growth was described by modeling the permeability of the endothelium as a function of wall shear stress (WSS). Tran-Son-Tay et al. (2007) and Tran-Son-Tay et al. (2008) investigated the problem by setting up an experiment using a rabbit model. Subsequently, they developed a mathematical model and compared it to the results obtained from the animal model, using a differential equation where the rate of change of the intima thickness is proportional to the shear stress. The model by Budu-Grajdeanu et al. (2008) includes cellular and chemical mechanisms and takes the change in lumen radius into account. Other studies (Dexter et al., 2009; Boyle et al., 2010; Hwang et al., 2011, 2013; Garbey and Berceli, 2013) developed agent-based models of the disease. Wu and Cassel (2013) modeled NIH as a diffusion process of smooth muscle cells using a feedback-control system, and simulated a reduction in NIH. Finally, in the model by Fok (2012), intimal thickening was modeled as a free boundary problem, which was an accurate description of cell and chemical dynamics, but did not include the flow characteristics which are an essential aspect in this study. A key feature of the work shown in this paper is that the simulations presented here are compared against patient-specific clinical data, which to the authors' knowledge, has not been done before in the context of NIH and bypass failure. Deidentified patient-specific data for this study was obtained with approval of the institutional human investigation committee (approval AD0009, Veterans Affairs Connecticut Healthcare System, West Haven, CT, USA) as part of an ongoing collaboration between UCL and Yale University.

This paper is organized as follows: the second section describes the methods used to develop the computational framework, including specific details about the different biological scales considered in the model, the mechanisms through which mechanical stimuli combine with biological changes, a description of how blood flow is modeled and how the two aspects of the model are combined in a framework. The third section presents specific simulation results for two vein grafts of a human patient and validation with clinical data. Finally, the fourth and fifth sections show a discussion of these initial simulation results and the conclusions, respectively.

This study is part of a broader research activity aimed at developing multi-scale, patient-specific models of cardiovascular disease (Alimohammadi et al., 2015; Di Tomaso et al., 2015), and in particular, vascular remodeling problems. A similar approach has been successfully implemented for the case of atherosclerotic disease (Díaz-Zuccarini et al., 2014). The patient-specific, multi-scale modeling framework proposed here uses a feedback approach to connect biological and mechanical mechanisms in vascular remodeling and details are provided below. In addition, this study seeks to establish the groundwork toward translational studies to understand the relationship between different multi-scale mechanisms and factors in patient-specific studies of vein graft failure, and to develop predictive tools for NIH-prone areas in vein grafts.

## 2. Methods

This paper brings together mathematical techniques and biological data to produce a model of NIH that describes the link between hemodynamic forces and significant biological mechanisms, according to the literature to date. The mathematical framework presented here relies on patient-specific imaging and hemodynamic measurements, which enables the possibility to study in detail the characteristics of the blood flow in patient-specific geometries. These data, together with literature findings (Humphrey, 2002; Budu-Grajdeanu et al., 2008; Duru et al., 2015), enable to identify areas most prone to NIH. In a nutshell, the process is as follows: patient-specific blood flow simulations on the vein graft prior to the formation of NIH (right after the procedure) are performed by means of a computational fluid dynamics software (ANSYS CFX). Subsequently, time averaged wall shear stress (TAWSS) results from the simulations are used as an input to a biochemical model of cell growth. This is mainly based on the association between WSS and the production of nitric oxide which has been established in previous research (Andrews et al., 2010), in addition to various other biological mechanisms, which will be explained in this section. A remodeling process occurs and a new geometry is then obtained. Finally, the steps are repeated until the time for comparison with clinical data is reached. Details are provided in the following sections.

### 2.1. Biochemical model

**Figure 1:**
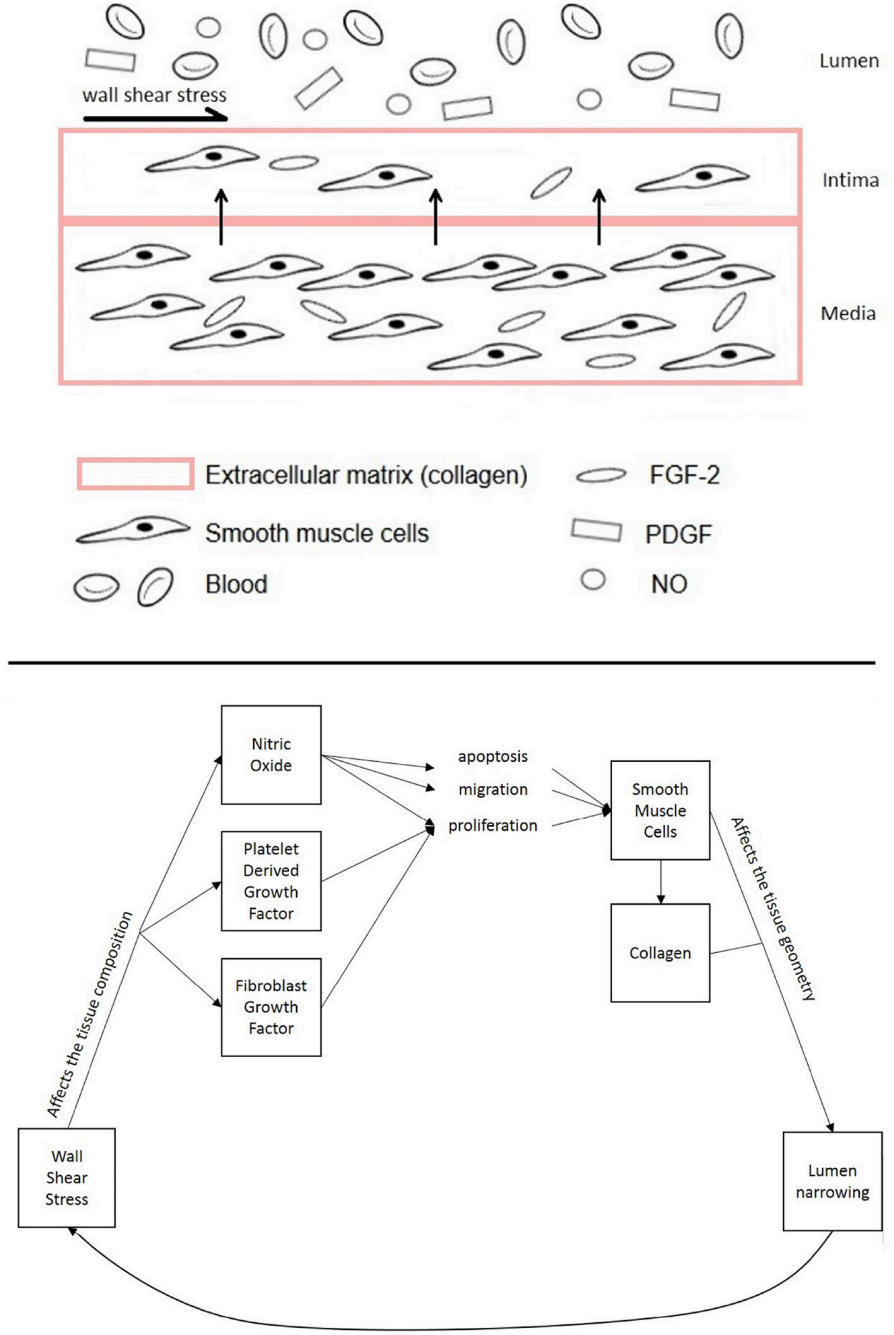
**Diagram of the different components of the vascular tissue accounted for in this model, showing the mechanisms affecting NIH progression**.

NIH occurs about 6–24 months after vascular intervention in the form of thickening of the tunica intima. This is one of the layers that constitutes the vascular tissue together with the tunica media and adventitia. While the adventitia is mainly composed of fibroblasts, the media and intima have very similar composition as they mostly contain smooth muscle cells. Normally, the tunica media is thicker than the tunica intima. However, after injury, smooth muscle cells, which are not normally characterized by high rates of proliferation, turn into a more synthetic type, with higher cell turnover and migration from the media to the intima, causing the vessel lumen to narrow (Figure 1). This causes the intimal volume to increase and often further surgical treatment is needed to avoid blockage (Collins et al., 2012).

This model considers smooth muscle cells proliferation in the intima to be the most critical response from vascular tissue after injury to the endothelium (Boyle et al., 2010; Model and Dardik, 2012). After injury, the start of the inflammatory process causes them to migrate from the media to the intima, accumulating there and causing the intima to thicken (Kohler, 2005; Model and Dardik, 2012).

The model allows for the calculation of intimal volume growth and lumen occlusion. The first process to affect smooth muscle cells in NIH is a change from the contractile phenotype, in which the production is very low, to a synthetic one with a much higher turnover (Collins et al., 2012). In the model proposed in this paper, the rate of production of quiescent cells (*Q*) is described by means of a logistic growth equation. The subscript *i* refers to the tunica intima, while *m* refers to the tunica media. The only inhibiting factor to the proliferation of the cells is the volume available to them, so the maximum numbers of cells *Q*_*i*_*max*__ and *Q*_*m*_*max*__ are calculated based on the maximum volume available in the intima and media, respectively. This approach to cell modeling has previously been used in mathematical models of tumor growth (Marusic et al., 1994; Kozusko and Bourdeau, 2007). Quiescent cells in the intima and media were modeled as

(1) $$\begin{array}{r} \frac{dQ_{i}}{dt}=\beta \times Q_{i}\times \left(1-\frac{Q_{i}}{Q_{i_{max}}}\right)\text{ in }\Omega_{i} \end{array}$$

(2) $$\begin{array}{r} \frac{dQ_{m}}{dt}=\beta \times Q_{m}\times \left(1-\frac{Q_{m}}{Q_{m_{max}}}\right)\text{ in }\Omega_{m}, \end{array}$$

with β = coefficient of quiescent cells turnover [day^−1^], *Q* = quiescent cells [cells], and *Q*_*max*_ = maximum number of quiescent cells [carrying capacity, cells]. Ω is function's domain, with Ω_*i*_ = tunica intima domain, and Ω_*m*_ = tunica media domain. Smooth muscle cells production is dependent upon phenotype change from quiescent cells, migration from the intima, presence of growth factors and production and apoptosis of cells that is in turn dependent on nitric oxide (NO). In the tunica media, they are assumed to be deriving from change in phenotype and cell production, and to degrade according to the amount migrating to the media and undergoing apoptosis. The coefficient of quiescent cells turnover β was estimated based on the assumption that the turnover rates in the intima and media are similar at a quiescent stage (Davies and Hagen, 1994).

(3) $$\begin{array}{l} \frac{dS_{i}}{dt}=\gamma \times Q_{i}+\;\left(\left(p_{i}-a_{i}\right)\times S_{i}+\;m\;\times S_{m}\right)\\ \;+\;\phi \times \left(G_{P}+G_{F}\right)\text{ in }\Omega_{i} \end{array}$$

(4) $$\begin{array}{rcl} \frac{dS_{m}}{dt} & = & \gamma \times Q_{m}+\left(p_{m}-a_{m}-m\right)S_{m}\text{ in }\Omega_{m}, \end{array}$$

with γ = coefficient of differentiation from quiescent cells [day^−1^], *p*_*i*_, *p*_*m*_ = coefficient of smooth muscle cell proliferation [day^−1^], *a*_*i*_, *a*_*m*_ = coefficient of smooth muscle cells apoptosis [day^−1^], *m* = coefficient of smooth muscle cells migration [day^−1^], ϕ = coefficient of production due to growth factors [cells/(ng × day)], *S* = smooth muscle cells [cells]. In the tunica media, *p*_*m*_ and *a*_*m*_ have been kept as proliferation and apoptosis coefficients as these are the values for standard conditions (high values of TAWSS) and the media was not considered to be affected by the TAWSS.

The collagen turnover is based on the approach proposed by Cilla et al. (2013) adjusting the parameter values according to the units used in the present model.

(5) $$\begin{array}{r} \frac{dC_{i}}{dt}=S_{i}\times \lambda -C_{i}\times \chi \text{ in }\Omega_{i} \end{array}$$

(6) $$\begin{array}{r} \frac{dC_{m}}{dt}=S_{m}\times \lambda -C_{m}\times \chi \text{ in }\Omega_{m}, \end{array}$$

with λ = coefficient of production from smooth muscle cells [g/(day × cell)], χ = coefficient of degradation [day^−1^] and *C* = collagen [g]. The response of smooth muscle cells to PDGF and fibroblast growth factor (FGF-2) based on WSS is modeled according to experimental data. This means that, although the general form of the equation is similar to that used in Budu-Grajdeanu et al. (2008), the equation's coefficients have been estimated from literature, to reflect the behavior of specific PDGF and FGF-2 growth factors.

(7) $$\begin{array}{r} \frac{d\left(G_{P}\right)}{dt}=\zeta_{g}-\zeta_{d}\times G_{P}\text{ in }\Omega_{i} \end{array}$$

(8) $$\begin{array}{r} \frac{d\left(G_{F}\right)}{dt}=\theta_{g}-\theta_{d}\times G_{F}\text{ in }\Omega_{m}, \end{array}$$

with ζ_*g*_ = PDGF production coefficient (ng/day), ζ_*d*_ = PDGF degradation coefficient (day^−1^), θ_*g*_ = FGF-2 production coefficient (ng/day) and θ_*d*_ = FGF-2 degradation coefficient (day^−1^). Table 1 reports the equations used to compute the parameters which vary with WSS. Finally, new volumes are calculated to find the growth of the tissue,

(9) $$\begin{array}{r} V_{i}=\left(S_{i}+Q_{i}\right)\times \rho_{s}^{-1}+C_{i}\times \rho_{c}^{-1} \end{array}$$

(10) $$\begin{array}{r} V_{m}=\left(S_{m}+Q_{m}\right)\times \rho_{s}^{-1}+C_{m}\times \rho_{c}^{-1}, \end{array}$$

with ρ_*s*_ = cell density [cells/m^3^], ρ_*c*_ = collagen density [g/m^3^] and *V* = volume [m^3^]. The model considers that cells have a uniform spatial behavior and the volume of vascular tissue is considered to be homogeneous. The tissue is divided in two layers, the tunica intima and tunica media, whose behavior is described by means of eight main equations. The volume of the tunica media remains constant as a result of the balance between apoptosis and proliferation of smooth muscle cells being steady, as this layer is not assumed to be affected by WSS. It has nevertheless been modeled to show the migration of smooth muscle cells from the media to the intima, and the turnover of cells in the tissue. Growth factors in the media have been disregarded. The biochemical species accounted for in the model are smooth muscle cells and collagen since they are the major constituents of the layers of vascular tissue considered (Humphrey, 2002). Tables 1, 2 summarize the model parameters and their respective values.

**Table 1:** **List of wall shear stress-dependent parameters**.

**Parameter**	**Definition**	**References**
WSS	Wall shear stress (Pa)	–
*R*_*NO*_	1.74+7.52 × *WSS* (M/s)	Andrews et al., 2010
*R*_*N*_*O*__*MAX*__	0.1 (μM/s)	Chen and Popel, 2006
*p*_*i*_	$p_{m}-p_{m}\times \frac{R_{NO}}{R_{NO_{MAX}}}$ day^−1^	–
*a*_*i*_	$a_{m}-a_{m}\times \frac{R_{NO}}{R_{NO_{MAX}}}$ day^−1^	–
m	$m_{0}-m_{0}\times \frac{R_{NO}}{R_{NO_{MAX}}}$ day^−1^	–
ζ_*d*_	1.94 × *WSS* × 10^−4^ day^−1^	Cilla et al., 2013
θ_*d*_	6.97 × *WSS* × 10^−4^ day^−1^	Cilla et al., 2013

**Table 2:** **List of constant parameters**.

**Parameter**	**Value**	**References**
β = turnover of quiescent cells	5 × 10^−4^ day^−1^	–
γ = rate at which quiescent cells become active	10^−4^ day^−1^	–
λ = collagen production	2.16 × 10^−13^ g/(day × cell)	Cilla et al., 2013
χ = collagen degradation	0.033 day^−1^	Cilla et al., 2013
ζ_*g*_ = growth rate (PDGF)	0.0776 ng/day	Palumbo, 2002
θ_*g*_ = growth rate (FGF-2)	0.1394 ng/day	Reisig and Clyne, 2010
*p*_*m*_ = smooth muscle cell proliferation coefficient	0.122 day^−1^	Poussier et al., 2005
*a*_*m*_ = smooth muscle cell apoptosis coefficient	0.0715 day^−1^	Poussier et al., 2005
*m* = smooth muscle cell migration coefficient	0.0251 day^−1^	Duru et al., 2015
ρ_*s*_ = cell density	2.18 × 10^14^ cells/m^3^	Schwartz et al., 1992
ρ_*c*_ = collagen density	2 × 10^3^ g/m^3^	Humphrey, 2002

Additionally the model describes the regulation of smooth muscle cells apoptosis, migration and proliferation through a direct relationship between WSS and shear stress-dependent biomolecules that regulate the vasculature. Numerous studies have shown that mechanical factors (e.g., WSS) influence cell response and thus the formation of NIH (Paszkowiak and Dardik, 2003; Caro et al., 2013; Tarbell et al., 2014; Jia et al., 2015). According to Humphrey (2002), NO, PDGF and FGF-2 play an important role in controlling smooth muscle cells turnover. While the relationship between growth factors and WSS has been modeled following the expression used in the study by Budu-Grajdeanu et al. (2008), multiple results from literature were considered when analyzing the relationship between WSS and NO (as described in the next section).

### 2.2. Relationship between WSS and NO

One of the ways cell production is influenced by WSS is through the relation between WSS and NO. When present near the vascular wall, a higher WSS causes a higher production of NO (Plata et al., 2010), which inhibits NIH (Ahanchi et al., 2007; Pearce et al., 2008).

Multiple models of NO production in response to WSS have been proposed using different approaches, for example, Chen et al. (2011) used a linear model, Fadel et al. (2009) used a hyperbolic model and Plata et al. (2010) a sigmolidal model, all of them derived from experimental data. The linear model was selected as it was shown in previous research (Andrews et al., 2010) to give results close to experimental values for shear stresses lower than 0.5 Pa, the threshold under which NIH is more likely to develop (Meirson et al., 2015). Nitric oxide production rate (R_*NO*_) was expressed as:

(11) $$\begin{array}{r} R_{NO}=1.74+7.52\times WSS, \end{array}$$

with *WSS* = wall shear stress.

The rate of production of NO has been shown to relate to the balance between proliferation, apoptosis and migration of cells. Studies have shown that low WSS, leading to a reduction of NOs, mRNA and protein expression, hinders the apoptosis signaling pathway, and induces platelet-derived growth factor (PDGF) and matrix metalloproteinase-2 (MMP-2) signaling pathway which leads to higher smooth muscle cells proliferation and migration (Qiu et al., 2013).

Experimental findings have shown NO production to be associated with the inhibition of cell growth in a linear (Marks et al., 1995; Nishio et al., 1996) or hyperbolic (Krick et al., 2002) way. In the particular case of the model presented here, linear relationships between NO production and the apoptosis (*a*), proliferation (*p*) and migration (*m*) coefficients are proposed:

(12) $$\begin{array}{r} a_{i}=a_{m}-a_{m}\times \frac{R_{NO}}{R_{NO_{MAX}}}, \end{array}$$

(13) $$\begin{array}{r} p_{i}=p_{m}-p_{m}\times \frac{R_{NO}}{R_{NO_{MAX}}} \end{array}$$

(14) $$\begin{array}{r} m=m_{0}-m_{0}\times \frac{R_{NO}}{R_{NO_{MAX}}} \end{array}$$

also reported in Table 1.

### 2.3. Patient data characteristics

**Figure 2:**
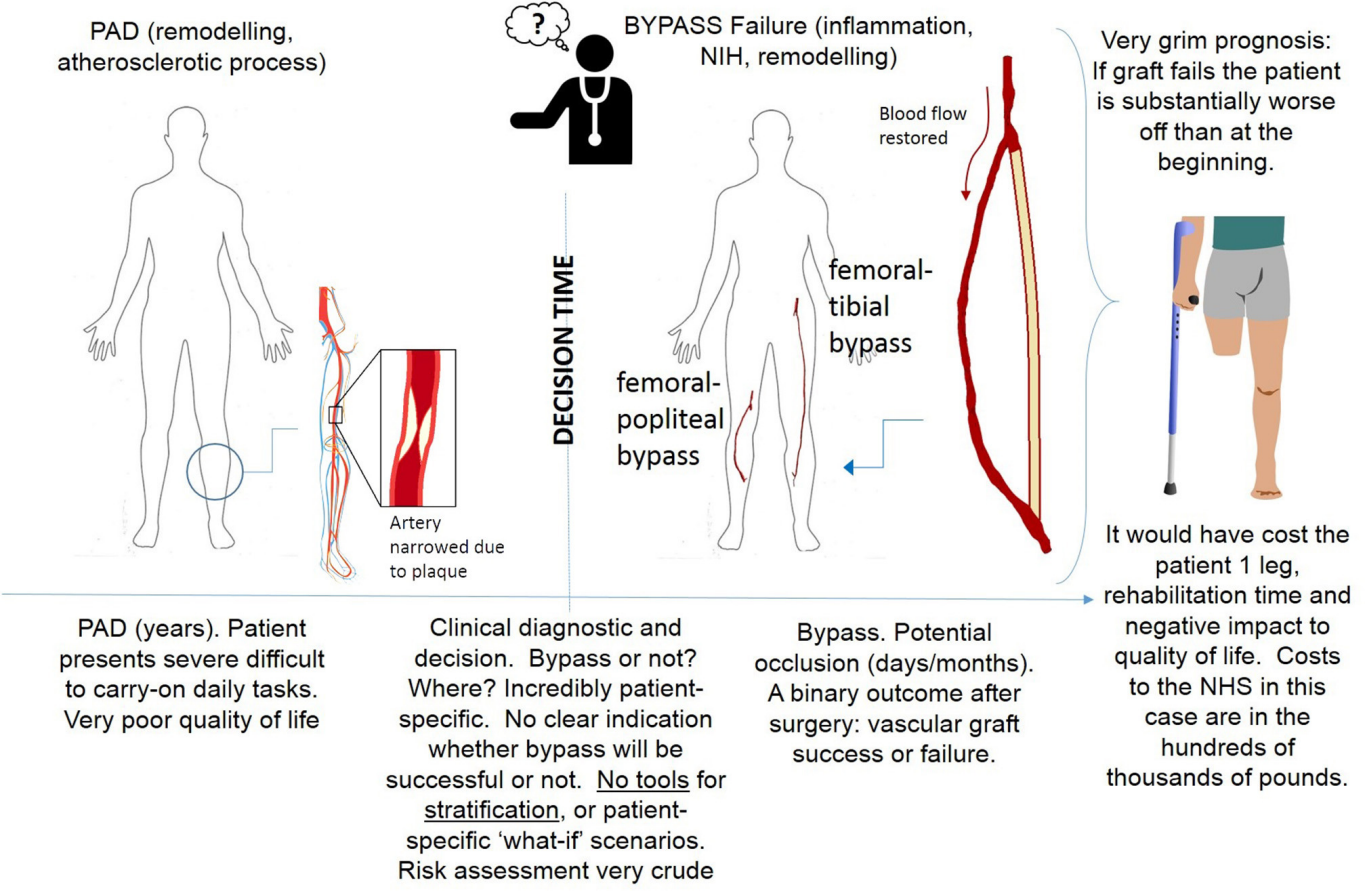
**Procedure leading to bypass surgery**.

Deidentified computed tomography (CT) scans were obtained from a patient that underwent bilateral peripheral vein graft bypasses procedures (approval from the institutional human investigation committee: AD0009, Veterans Affairs Connecticut Healthcare System, West Haven, CT, USA); in the right leg a femoro-popliteal bypass was performed from the mid-thigh to the mid-calf, and in the left leg a femoro-distal bypass was performed from the groin to the mid-calf. A diagram of the procedure leading to the implantation of vascular grafts is shown in Figure 2. Doppler ultrasound and noninvasive pressure measurements were also taken at different locations along both legs. All studies were performed as part of the standard clinical care for the patient after surgery. Consent for the studies was obtained.

### 2.4. Image processing

Images of the patient-specific arterial geometries were extracted from the CT scans using ScanIP (Simpleware Ld., Exeter, UK). The post-operation images were processed using ScanIp to obtain two different 3D geometries for each of the bypasses (right leg and left leg): one immediately after surgery and another a few months after surgery (4 months for the right leg and 8 months for the left leg bypass). While the geometries a few months after surgery were part of the clinical dataset, the geometries immediately after surgery were not available within the dataset, and were obtained by locating the areas where NIH had formed as exemplified in Budoff and Shinbane (2010), and virtually removing them. The right side graft had an initial radius of 2.72 mm at the inlet, while the outlet radius measured 2.67 mm. The length of the selected volume for the analysis (from inlet to outlet) was 30 cm. The left side graft had a more complex geometry due to the presence of multiple outlets, the greater length of the graft itself, and the difference in dimensions between different parts of the geometry. The radii of inlet and main outlet were 6.29 and 0.96 mm, respectively, while the outlets of the peripheral vessels measured 2.71 mm (deep anterior femoral), 1.63 mm (deep posterior femoral), 1 mm (posterior distal—proximal outlet). The length of the volume considered for the left side graft was 90 cm.

### 2.5. Computational fluid dynamics model

A commercial software, ANSYS CFX 17 (Ansys Inc, PA, USA) which is based on Finite Volume Methods was used for CFD simulations. The Navier Stokes equations were spatially discretized using a high resolution upwind scheme. A second order implicit backward formula (called second order backward Euler scheme by the software) was used for the temporal discretization (Ferziger and Peric, 2013); the time-step size was 0.0025 s. Blood was assumed to be an incompressible, homogeneous and Newtonian fluid. Blood characteristics were a density of 1,050 kg/m^3^ and viscosity of 0.0035 Pa·s. For each of the simulations in the remodeling cycle, an unstructured mesh of 400,000 elements was created, each with 7 prismatic layers. A grid sensitivity analysis was carried out for the first simulation on a transient case to compare values of TAWSS. A 7% difference in TAWSS was found between the 400,000 elements and 1M elements meshes. The difference in velocities at the outlet was less than 2%. This was considered enough for mesh convergence. The same approach to meshing was used for the left leg bypass geometry, which led to finding an optimum number of elements at 2.9 million elements. It is worth noticing that the mesh for the left leg graft had an increased number of elements, partly due to the fact that the simulated section is significantly extended since femoro-distal bypasses run along the whole leg (Figure 2), but also to the fact that the very small collateral vessels were also meshed and this required an appropriate element size.

### 2.6. Boundary conditions

**Figure 3:**
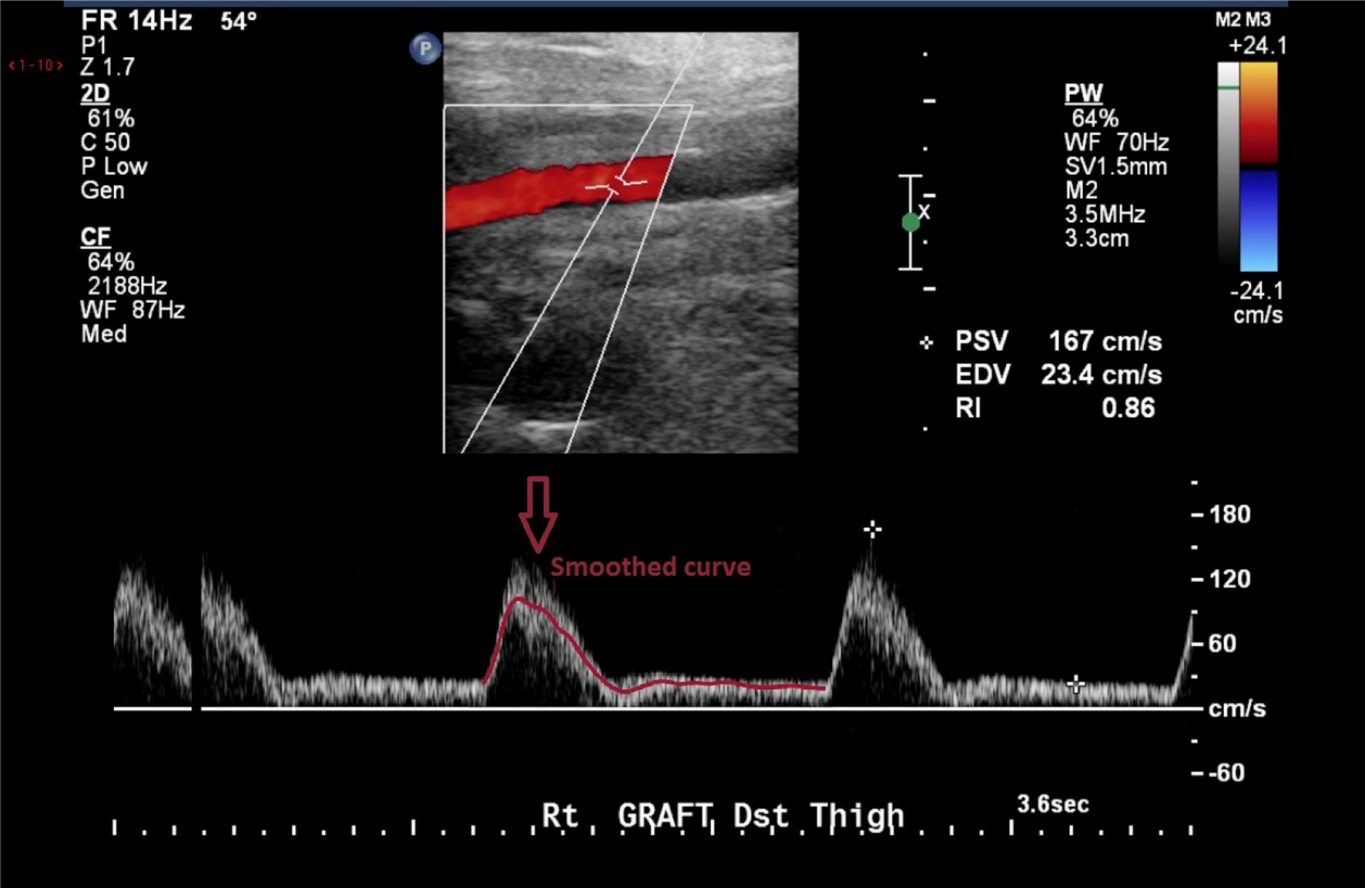
**Doppler images from the clinical dataset had to be processed in order to remove unphysiological peaks**.

At the inlet, a pulsatile parabolic velocity boundary condition was applied. This was obtained from the Doppler ultrasound data available as part of the clinical dataset. The curves were smoothed in Matlab (The MathWorks Inc., Natick, MA, USA) to avoid non-physiological oscillations and used to compute mass flow rate curves. The standard Matlab smooth function based on a moving average filter was used to smooth the data. An example of the level of smoothing applied to the images can be seen in Figure 3. The ultrasound measurements obtained from the clinical dataset presented quite a significant amount of noise which had to be removed, as shown in the figure. A velocity profile at the inlet of the flow domain was then obtained from the estimated mass flow rate at the location of the ultrasound measurement and used for creating a parabolic profile according to the equation (Munson et al., 2002):

(15) $$\begin{array}{r} u\left(r\right)=V_{max}\times \left(1-\frac{r^{2}}{R_{max}^{2}}\right), \end{array}$$

where *u*(*r*) = velocity profile along the radius, *V*_*max*_ = maximum velocity curve from Doppler data, *r* = position along the lumen radius, *R*_*max*_ = lumen radius.

#### 2.6.1. Right leg

For the right leg graft, which featured only two openings (an inlet and an outlet), a zero pressure-gauge boundary condition was used at the outlet, with no further pressure or boundary conditions. For a rigid wall model with no bifurcations, this is considered to be a reasonable approximation (Wood et al., 2006). Four cardiac cycles, each 1.1 s long, were simulated to reach periodic steady-state.

#### 2.6.2. Left leg

**Figure 4:**
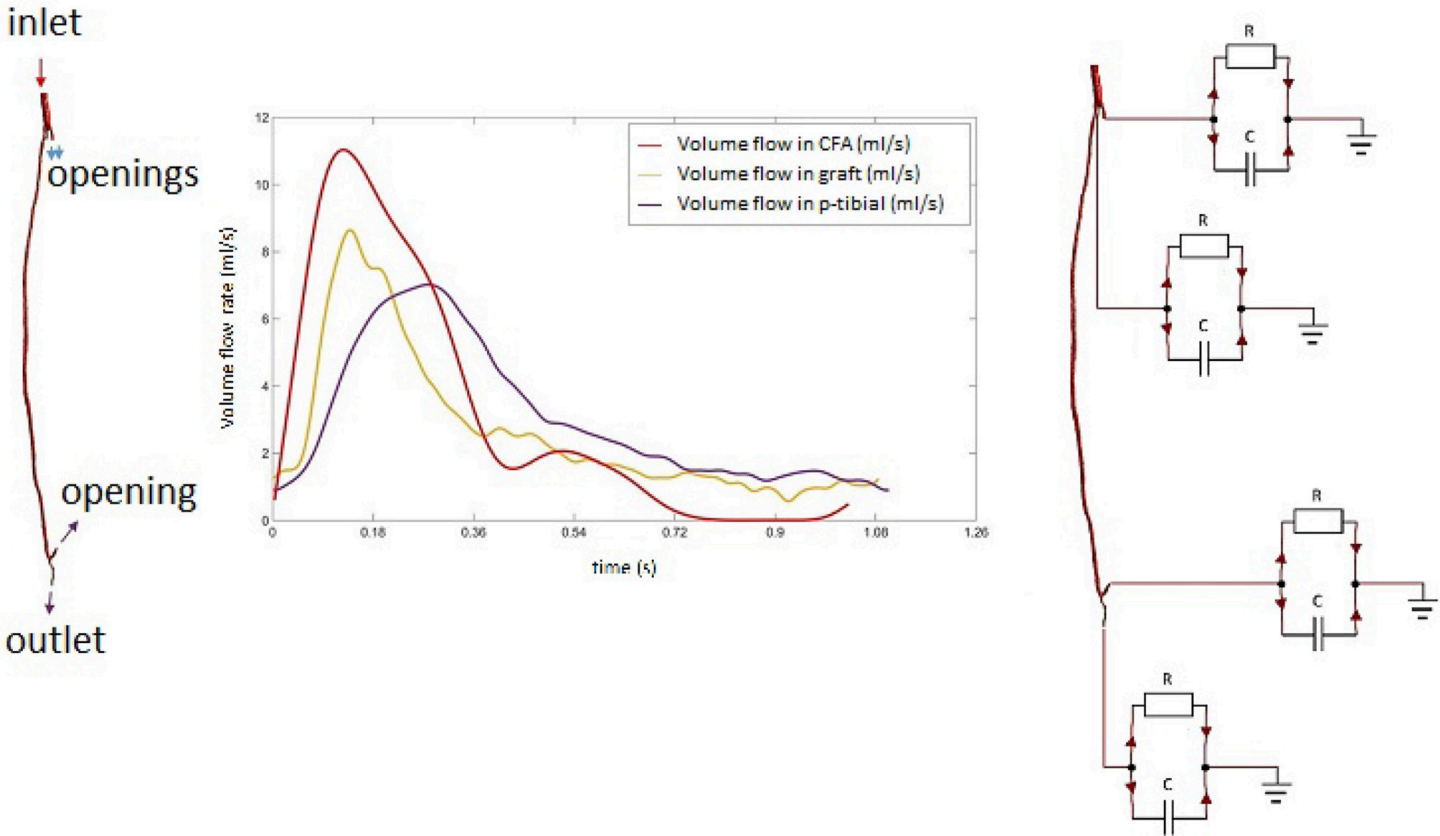
**Mass flow rate profiles in the common femoral artery with diagram of the multi-scale (0D-3D) model**.

The left leg bypass featured five different openings, of which one inlet and four outlets. The presence of the four outlets was confirmed by estimating the flow difference between the graft and the common femoral artery. This was done with the help of Doppler data provided as part of the clinical dataset. The velocity curves were extracted from the Doppler ultrasound images for the common femoral artery, the graft and the popliteal artery. Mass flow rates were calculated for each of the vessels. Collateral vessels were modeled as openings following a mass-conservation analysis (Figure 4). In the case of multiple outlets, lumped parameter models have been shown to provide a good estimate of the conditions at boundaries allowing to relate pressure to flow through the use of resistance, capacitance and inductance parameters (resistance, compliance and inertance the hydraulic equivalent) which are based on the analogy of fluid systems to electrical circuits (Westerhof et al., 2008; Alimohamadi et al., 2015). In order to estimate model parameters for peripheral resistance and compliance, a lumped parameter model of the vasculature was built using 20-sim (Controllab Products, Enschede, the Netherlands). The 3-D domain was simplified by modeling segments of the geometry with corresponding values for resistance and inductance parameters as seen in Westerhof et al. (1969). This model was used to test and adjust parameters for arterial resistance and compliance to adapt them to the geometry specific to this case. To calculate the resistance, mean pressure difference over mean flow (both part of the clinical dataset) was evaluated to get an estimate as a starting point for finding the appropriate values, and compliance was computed starting from values found in the literature (Stergiopulos et al., 1992). Using the starting parameters (from the literature) for resistance and compliance, the 0-D model was run in 20-sim to obtain flow curves at the outlets. These were compared against the curves from clinical data using the hydraulic-electrical analogy. Changing the values of resistance and compliance parameters at the outlets allowed to adjust the flow curves obtained via 20-sim until matching those from the clinical dataset. A summary of the final parameters used is shown in Table 3. After this first calibration, the 0D and 3D models were coupled using Ansys CFX 17 (Ansys Inc., Canonsburg, PA, USA). A diagram of the lumped parameter model and the application of boundary conditions to the 3D model is shown in Figure 4.

**Table 3:** **List of parameters used in the 0D model**.

**Parameter**	**Value**
C = capacitance	12.6 × 10^−6^ ml/Pa
R (deep femoral artery - anterior) = resistance	5.5 × 10^5^ Pa s/ml
R (deep femoral artery - posterior) = resistance	4.5 × 10^5^ Pa s/ml
R (deep femoral artery - posterior) = resistance	4.5 × 10^5^ Pa s/ml
R (popliteal artery - anterior) = resistance	3 × 10^3^ Pa s/ml
R (popliteal artery - posterior) = resistance	2 × 10^3^ Pa s/ml

### 2.7. Remodeling cycle

**Figure 5:**
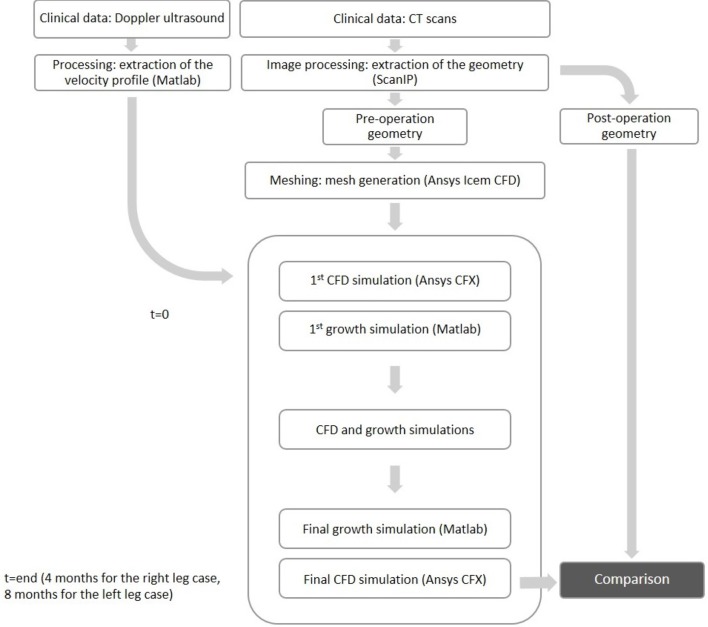
**Flowchart of the NIH remodeling process used in this study**.

In order to simulate the occlusion of the vessel based on the flow characteristics and biochemical processes considered, a remodeling cycle was used to combine the two approaches in such a way that the biochemical model could feed back into the fluid dynamics model and conversely information on WSS would inform the biochemical model (Figure 5). After the first CFD simulation, results were obtained for TAWSS at each mesh node. These values were used to calculate tissue growth at each node by means of the biochemical model discussed above. As it was not possible to run enough cardiac cycles (on a timescale of seconds) in the CFD model to cover the whole timespan of disease development (on a timescale of months), an assumption had to be made to couple the two timescales while remaining within a reasonable computational time. The CFD model was run for 4 cardiac cycles at a time-step size of 25 ms, which allowed to reach periodic steady state. After obtaining the CFD results, the biochemical model was run using the results for wall shear stress from the previous CFD simulations, time averaged over one cardiac cycle in order to capture the variation of the variable over time. A new geometry file accounting for the growth was then created using the results from the biochemical model. This was run with a time-step size of 1 day, either for the full length of time from surgery to clinical data acquisition (in the case of the left leg bypass), or until reaching a significant change in geometry (after 2 months, in the case of the right leg bypass). Finally, a new CFD simulation was run to obtain values of TAWSS and other hemodynamics indicators to model the flow in the new lumen geometry, and the cycle either finished or repeated in case the total time had not yet been achieved.

At the end of the remodeling cycle, results were obtained for comparison with clinical data. The analysis was carried out using ScanIp (Simpleware Ld., Exeter, UK) and Ansys CFD-Post (Ansys Inc., Canonsburg, PA, USA). The lumen occlusion was computed according to the formula below.

$$\begin{array}{l} \text{Percentage of lumen volume occluded}=\\ 100\times \frac{\text{Initial cross sectional area – Final cross sectional area}}{\text{Initial cross sectional area}} \end{array}$$

## 3. Results

**Figure 6:**
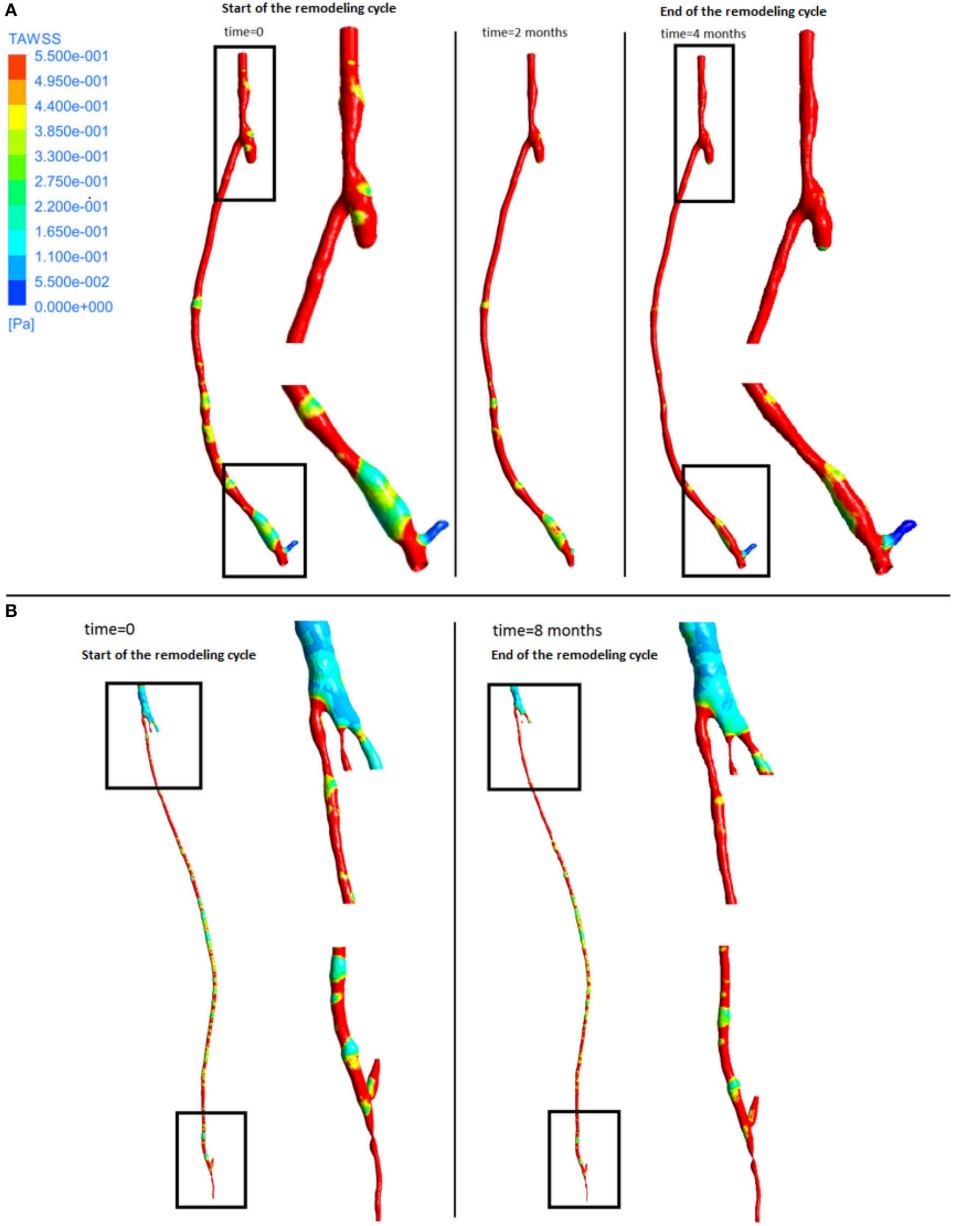
**Time average wall shear stress at different stages of the remodeling cycle in the right leg graft (A)** and left leg graft **(B)**.

**Figure 7:**
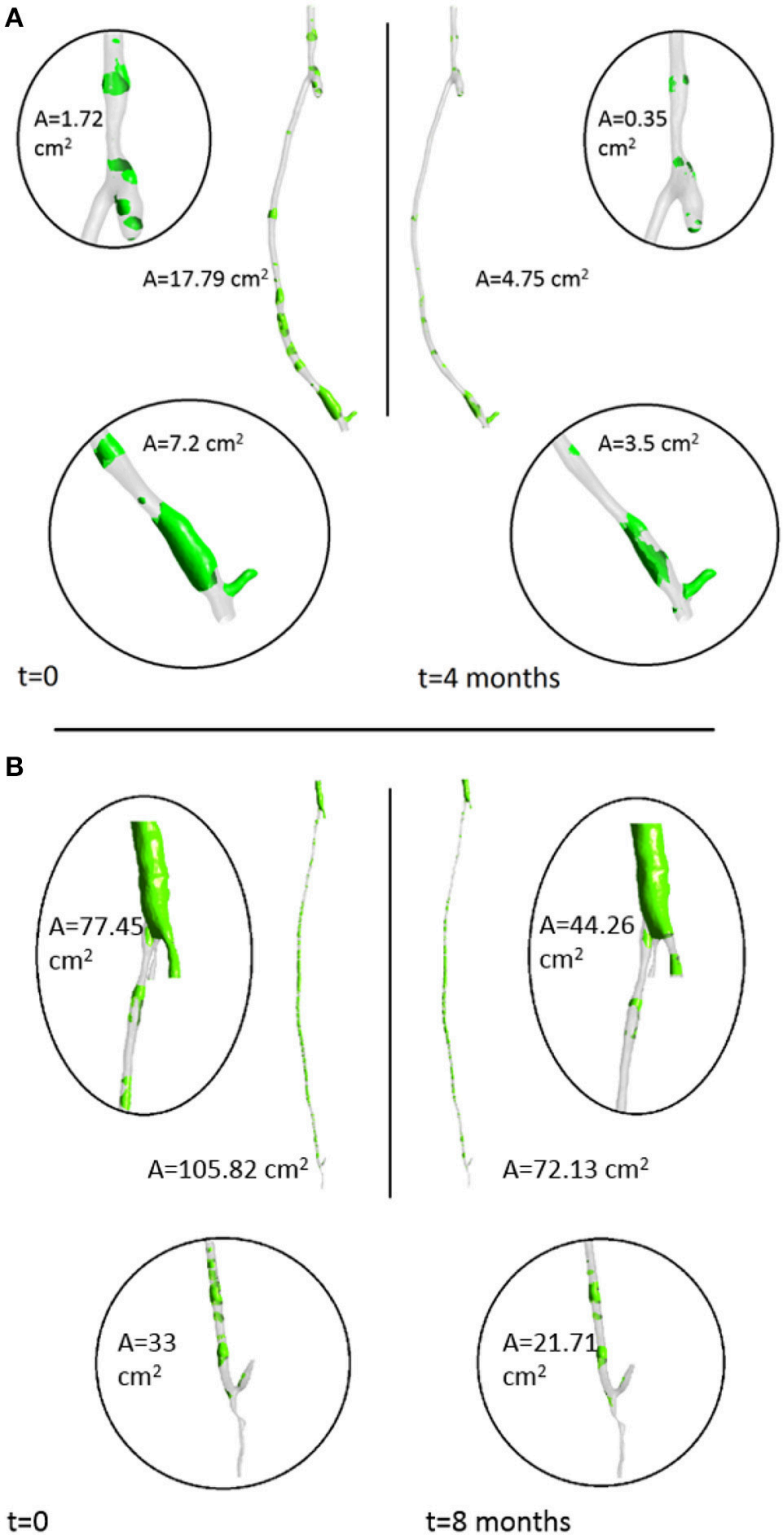
**Areas of low TAWSS (<0.5 Pa) in the right leg graft (A)** and left leg graft **(B)**.

**Figure 8:**
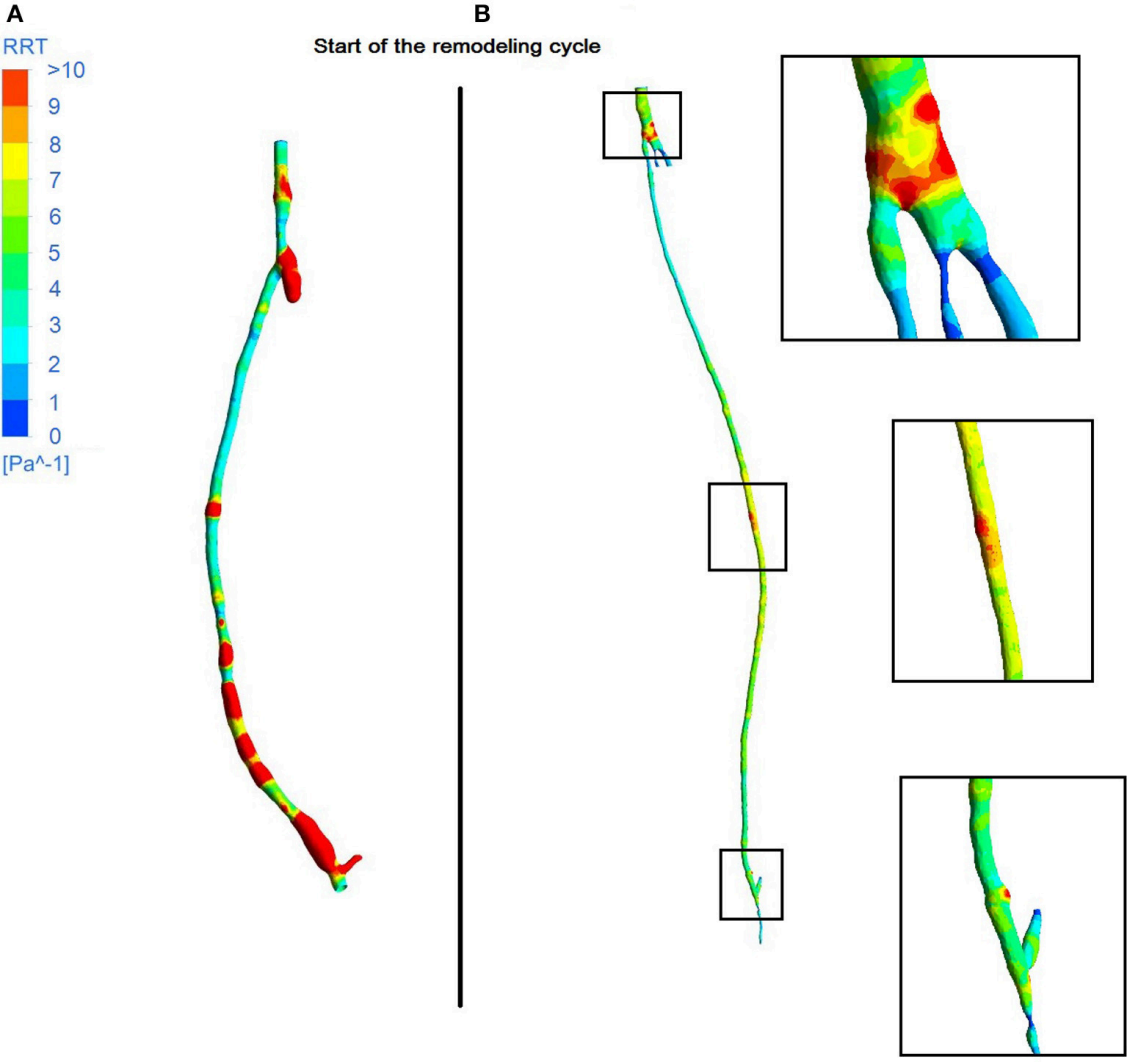
**Contour plots of RRT in the right leg graft (A)** and left leg graft **(B)**.

From the fluid dynamics simulations, the variable of interest for the model is TAWSS, as this is the mechanical factor that has an influence on the turnover of cells. Results of TAWSS were extracted after each step of the remodeling cycle, in order to be used as input data for the biochemical model. Figure 6A shows TAWSS in the right side graft at the beginning of the cycle, and after 2 and 4 months, while contour maps of the TAWSS values in the left side graft at time zero and at 8 months are shown in Figure 6B. It can be noticed that in both cases WSS distribution changes as the remodeling occurs, and that low TAWSS areas tend to be located in the proximity of bifurcations or turns (as shown by the zoomed in areas in the figure). For instance, in the right side graft case, the areas where TAWSS is lowest are at the proximal and distal anastomosis, with the latter showing a much more extended area where TAWSS is less than 0.5 Pa. Zones with low TAWSS are also present along the graft. However it is interesting to note that due to the remodeling process these areas tend to decrease when not situated near parts of the graft where the flow is likely to be deflected. The same is also valid for the left side graft, in which low TAWSS is located where the diameter of the graft is exposed to sudden changes, or before bifurcations. To illustrate the importance of the remodeling of the graft and how it changes the WSS distribution, a comparison between TAWSS contour plots and hyperplasia growth was made (Figure 7). After the remodeling, the changes in the geometry of the graft have a significant impact on the hemodynamics, which in some cases reduced the areas of low TAWSS, while in others it further reduced TAWSS thus causing the area to become even more prone to growth. By affecting the geometry, the locations of NIH growth have a significant impact on the next remodeling cycle. To illustrate the importance of remodeling in the graft, the areas of critical TAWSS (<0.5 Pa) were measured before and after the full remodeling cycle (Figure 7). In the right side graft, this showed that the remodeling led to a decrease in the areas of critical TAWSS with the total area dropping from 17.79 to 4.75 cm^2^. However, the area close to the distal anastomosis changed more slowly from 7.2 to 3.5 cm^2^, so the extent of low TAWSS remained significant, causing a critical amount of growth in the area near the distal part of the graft. This part of the graft was also where the lowest values of TAWSS were found, with TAWSS as low as 0.2 Pa before remodeling took place. On the other hand, the proximal part of the anastomosis was subject to a change from 1.72 to 0.35 cm^2^ of the low TAWSS area, which, while still causing some degree of growth, did not have such an impact as in the distal segment. A similar behavior was observed in the left graft, with the total area of low TAWSS decreasing from 105.82 to 72.13 cm^2^, the area near the proximal anastomosis from 33 to 21.71 cm^2^, near the distal anastomosis from 77.45 to 44.26 cm^2^. Both grafts present a curvature toward the end, which has an effect on the velocity profile. When going through a turn, the flow at the center for inertia moves slower than the flow near the wall, causing it to move away from the center of the curvature (Giordana et al., 2005). This results in a different flow pattern compared to the case for a straight artery, leaving space for the formation of secondary flows and also slowing down the main flow, both of which are factors causing lower levels of shear stress. For further analysis, the relative residence time (RRT) index, which has previously been used for other cardiovascular diseases to identify areas where TAWSS was low and oscillatory shear index was high, for instance in the study of atherosclerosis (VanderLaan, 2004), was also considered. As shown in Figure 8, this index also indicates the locations of the most severe hyperplasia progression, which suggest high oscillatory shear stress might also play an important role in the development of the disease.

**Figure 9:**
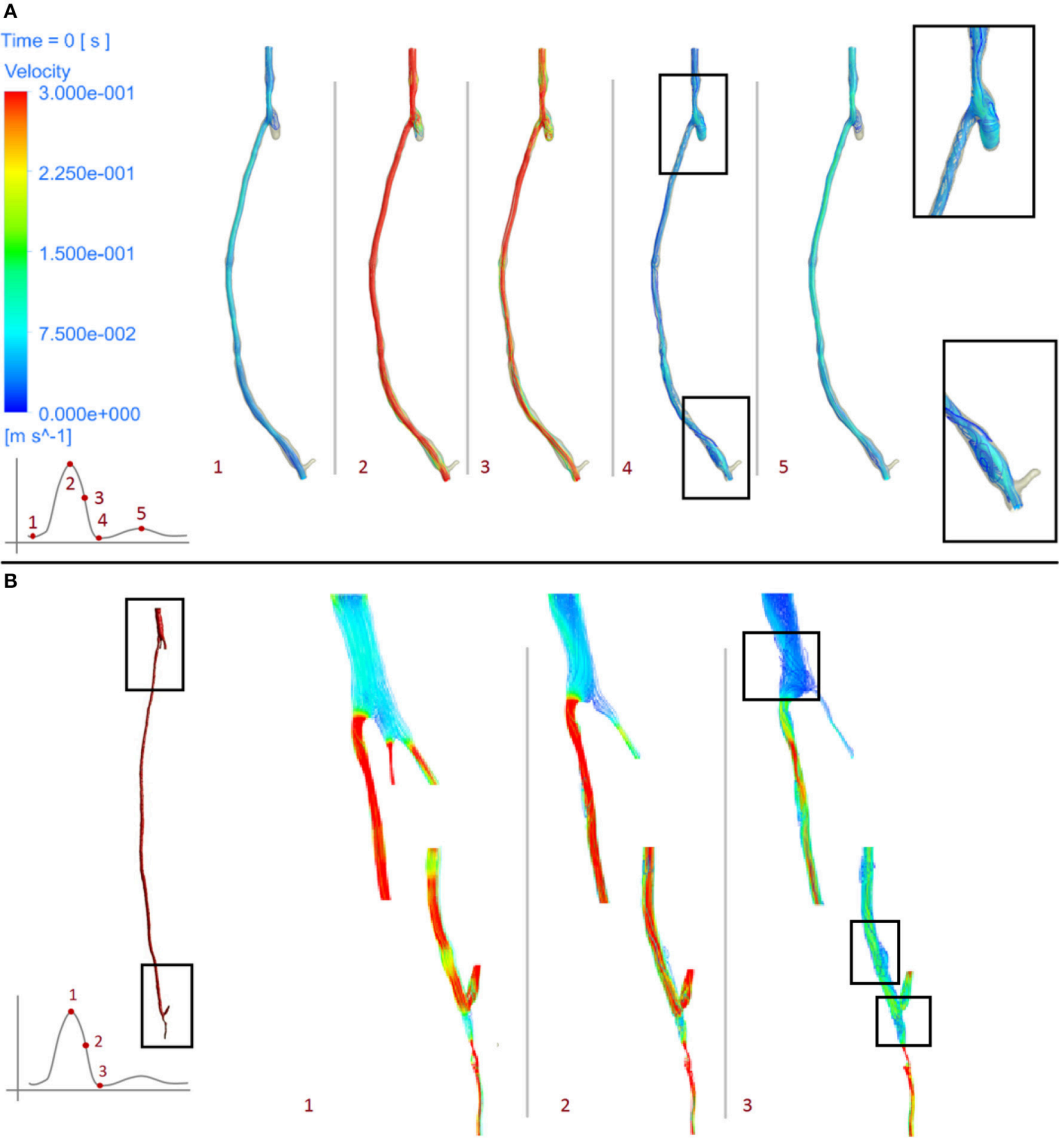
**Velocity streamlines at different stages of the cardiac cycle of the right leg graft (A)** and left leg graft **(B)**.

As can be seen from the streamline plots in Figure 9, low TAWSS is located mostly in the proximity of areas where the flow is disturbed due to the presence of bifurcations. In the right side graft, at the onset of systole (point 1), flow was steady and some recirculation zones were observed only after the proximal anastomosis, due to the presence of the occluded vessel. At peak systole (point 2), the velocity was uniform although it decreased at the proximal anastomosis and through the slightly larger diameter of the cross section near the distal anastomosis. As the flow slows down and goes into the diastolic phase (points 3, 4, 5), a different distribution can be seen, with a disturbance of the flow at the distal anastomosis, which will likely cause lower values of WSS, especially where changes in diameter occur. This agrees with previous research showing changes in the intensity of vortices after the flow passes from the graft to the artery (Doorly et al., 2002). A similar behavior can also be noticed in the left side graft, where the flow is more streamlined when at peak systole (point 1), except after the narrowing distal to the bifurcation at the lower end, where some reversed flow can be noticed even at systole. When the flow slows down and goes into diastole (points 2, 3), in addition to separation and reversed flow especially before and after bifurcations and where the graft joins the artery, some helicoidal flow can be seen right after the very narrow section at the distal location.

**Figure 10:**
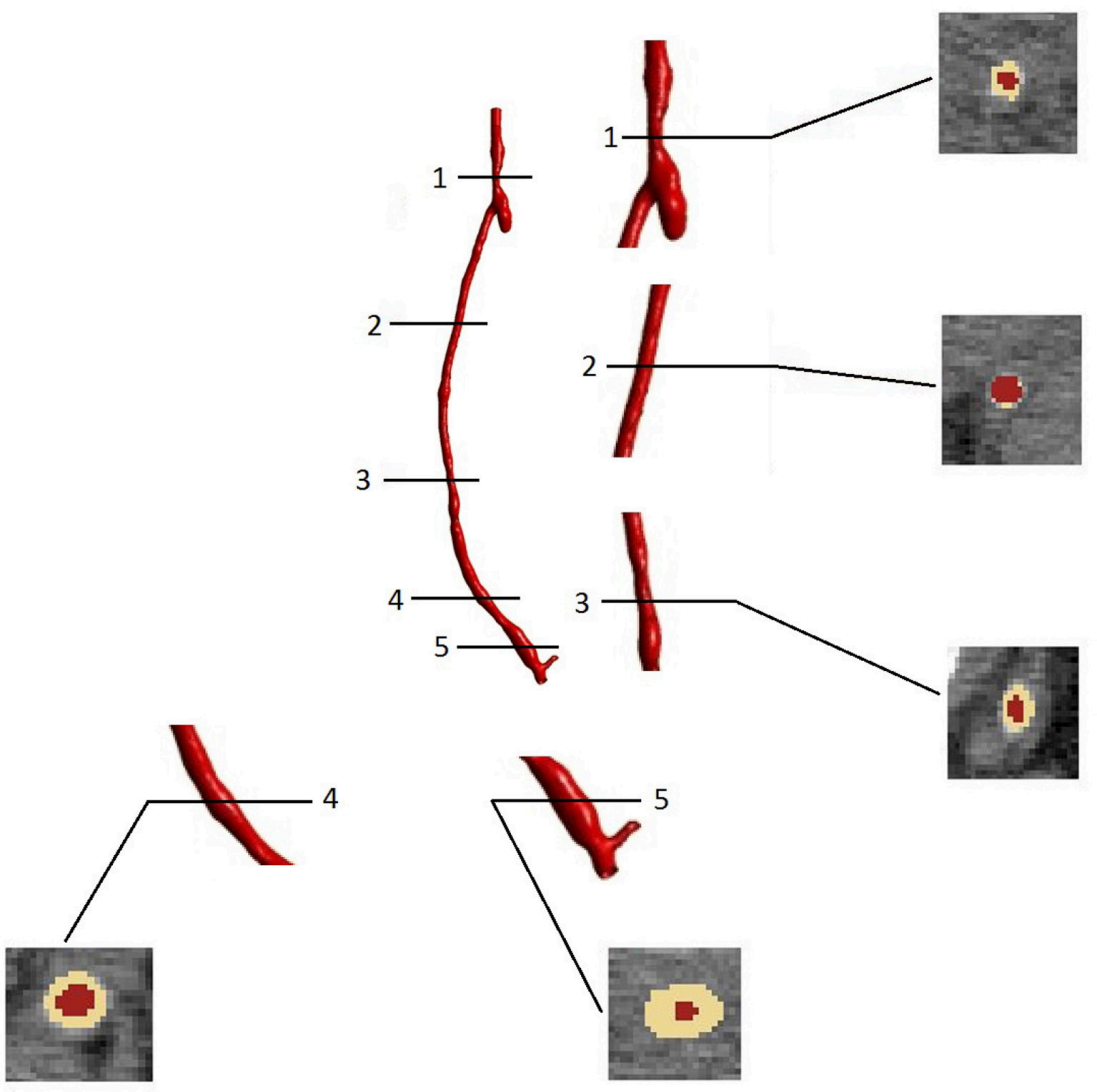
**Cross sectional areas at different points of the bypass grafts (after 4 months of NIH progression) visualized in the image processing software ScanIp (Simpleware Ld., Exeter, UK)**.

**Figure 11:**
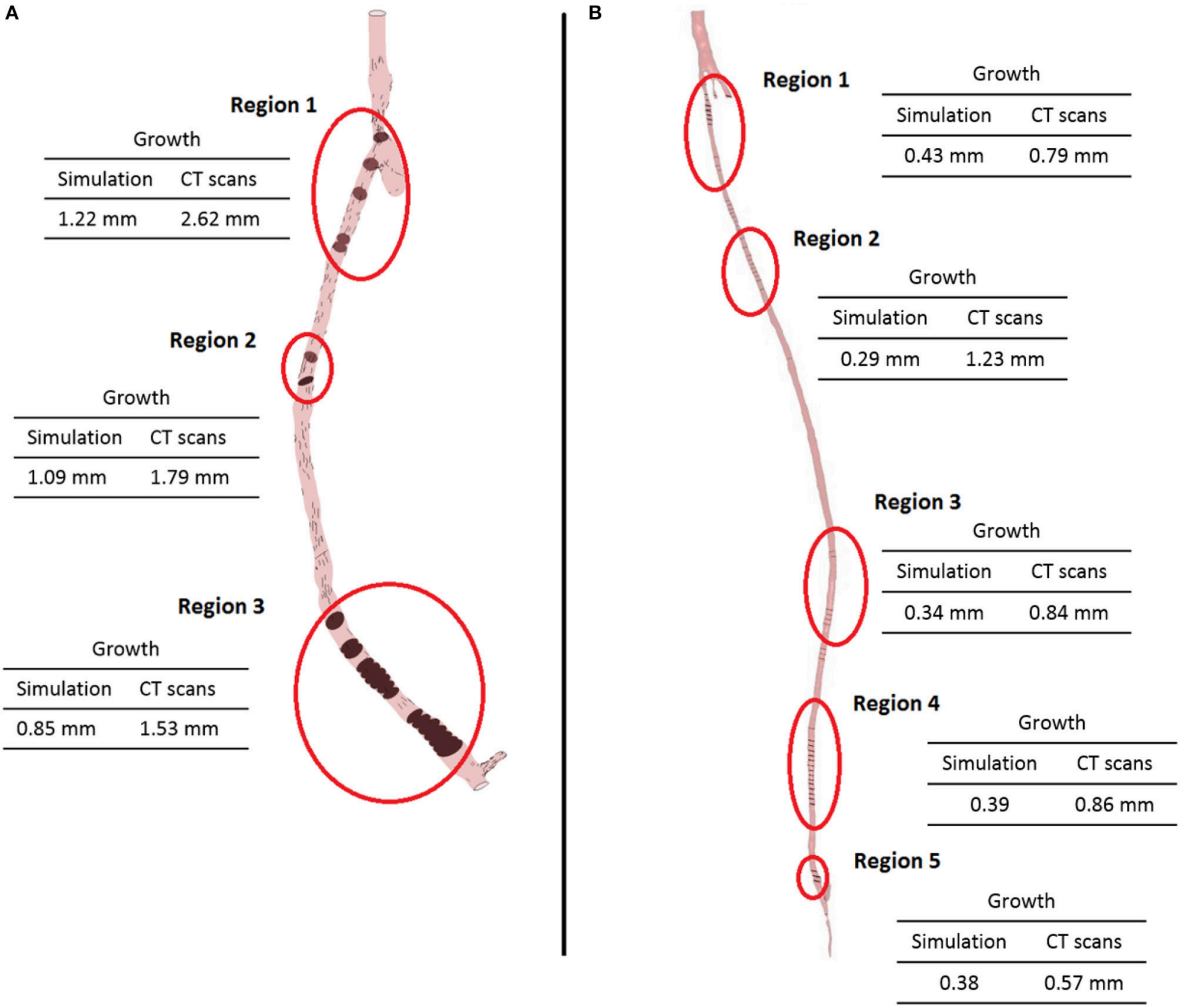
**Locations of the most critical zones of NIH development (circled) in the right leg graft (A)** and left leg graft **(B)**.

As expected, the disease progression had a different impact at different locations along the graft. Figure 10 shows five different cross sections of the right hand side bypass with their respective lumen occlusion as obtained from the radiological images. The lumen geometry at time zero is shown in pale yellow, whereas the geometry at 4 months post-operation is marked in dark red. As can be seen, the disease progression was different for the cross sections selected, with most hyperplasia forming in proximity of the anastomoses. Figure 11A shows the locations where the most critical values of NIH development were found in the CT scans in the right bypass, while the critical locations on the left bypass are shown in Figure 11B. As restenosis is defined as the development of 50% or more luminal narrowing in the graft (Hill, 2004), this was chosen as the threshold to select the locations of critical NIH development. The measurements show that the locations of growth were reproduced by the model.

## 4. Discussion

As mentioned in the introduction, several computational approaches have been proposed in the literature to model NIH or restenosis. While some studies focused on developing agent-based models (Dexter et al., 2009; Boyle et al., 2010; Hwang et al., 2011, 2013; Garbey and Berceli, 2013), others (Budu-Grajdeanu et al., 2008) used a deterministic approach based on ordinary differential equations. More recently, the increasing use of computational modeling in medical applications and the latest advancements in these techniques have allowed to study the disease from different angles. Some studies have been using CFD as a tool for analyzing the behavior of the flow in pathological cases. In many cases, studies have been conducted to analyze the flow in stents and grafts and to build hypotheses on its effect on NIH and restenosis. This has confirmed the hypothesis of low shear stress and abnormal flow patterns leading to NIH in the pulmonary artery (Berdajs et al., 2015), carotid artery (Harrison et al., 2014), coronary arteries (Guerciotti et al., 2016) peripheral arteries (McGah et al., 2012; Rivera et al., 2014). In addition, some studies have focused on specific applications, such as vascular access for hemodialysis, looking at minimizing NIH by finding the optimum conditions for performing arteriovenous fistulae operations. Research has been carried out in this area on hemodynamic factors such as WSS (Jia et al., 2015) and oscillatory shear index (Ene-Iordache et al., 2015), but also on variables such as blood flow rate and needle tip position (Fulker et al., 2016). CFD has also been used to analyze possible design optimization routes for grafts for both hemodialysis (Canneyt et al., 2013), coronary artery stents looking at the flow within stent struts (Gundert et al., 2012), comparing different stent geometries (Gogas et al., 2014) and the effect of deformable stents (Martin et al., 2014), peripheral bypasses (Grus et al., 2016), and novel grafting systems such as an endograft for aneurysm repair (Aristokleous et al., 2015) and a mechanism of external vein graft support (Meirson et al., 2015). Moreover, new studies looking at the interaction between mechanical forces and cell response for instance used agent based (Ziraldo et al., 2013), hybrid agent based-continuum (Garbey et al., 2015), coupled agent-based and finite element (Zahedmanesh and Lally, 2011), and mechanistic approaches (Goodman et al., 2016).

The model of NIH presented in this paper shows an intra-patient comparison of two different bypass geometries and the applicability of a patient-specific, multi-scale approach to each case. The physiological parameters used by this model have been tested in previous studies to quantify the behavior of SMC and collagen and its influence on NIH progression. The ability of the model to produce results and to formulate and test hypothesis on a macroscopic scale using data from experiments conducted on a cellular level shows the applicability of a quantitative approach to the interpretation of biological data and processes. The model confirmed that the behavior of the biochemical species play an important role in the disease progression. The model validation was carried out against a patient-specific, unique clinical dataset by detecting the locations of restenosis in the CT scans from the patient, and then comparing them against the simulated results. As can be seen in Figure 11, the locations of restenosis in the patient corresponded to those estimated by the model.

In the right side graft, growth estimated for each point ranges from 0.85 mm (region 3) to 1.2 mm (region 1) whilst in the CT scans, an approximate growth of 1.5 mm (region 3) to 2.6 mm (region 1) is observed. In the left side graft, values of growth at the critical locations measured 0.3–0.4 mm in the simulated geometry, while in the real case were estimated to be around 0.8 mm. It is however difficult to correctly measure NIH development due to the quality of the CT scans and uncertainty in the measurements. Additionally, some sections of the artery occluded in the real case and although the location was relatively well predicted, growth was underestimated by the model. There is a complex interplay between the remodeling/growth in the model and the hemodynamic parameters and this warrants further investigation.

This shows that combining a model describing biochemical interactions and a mechanical model describing the hemodynamics is a promising approach to describe the behavior of the patient's disease. This type of model has a potential use as a clinical tool to inform clinicians on the progression of the disease, and future modifications will allow not only to locate NIH but also to inform on the severity of the growth. The applicability of the model to patient-specific geometries was also evaluated on two different types of vein graft, for both of which the model located areas with the most NIH development. In addition, the study also lead to a further analysis of the importance of including collateral vessels in the model, which were found to have an influence on the fluid dynamics results and consequently on the biochemical processes as well.

This work has shown the potential of using experimental data to develop integrative *in silico* models to study NIH progression. A combination of data coming from *in vivo* and *in vitro* experiments was particularly useful to get a better understanding of the disease and design a computational model able to produce quantitative information related to relevant dynamical processes of NIH progression. In particular, from the fluid dynamics aspect it was important to be able to apply an appropriate type of boundary conditions. Although for the right side bypass this was relatively straightforward due to the presence of only one inlet and one outlet and the validity of the stress-free boundary condition at the outlet, the conditions were more complicated on the left graft. As stated above, boundary conditions for this model were provided by a lumped parameter model. This allowed to account for the effects of resistance of the peripheral vessels, through the resistance parameter, as well as vessel elasticity through the compliance parameter (Shi et al., 2011). Applying a zero-pressure boundary condition in this case would have led to a non-physiological flow split, with most of the flow being directed toward the path of least resistance. In addition, accounting for these aspects of the flow allows to obtain physiological pressure curves (Kim et al., 2010).

Finally in order to study the performance of the whole multi-scale modeling approach proposed in this paper, the sensitivity of NIH development with respect to key parameters in the biochemical model was evaluated. In this initial analysis the kinetic rates related to proliferation and apoptosis of smooth muscle cells were selected (given the key role of these cells in NIH progression).

**Figure 12:**
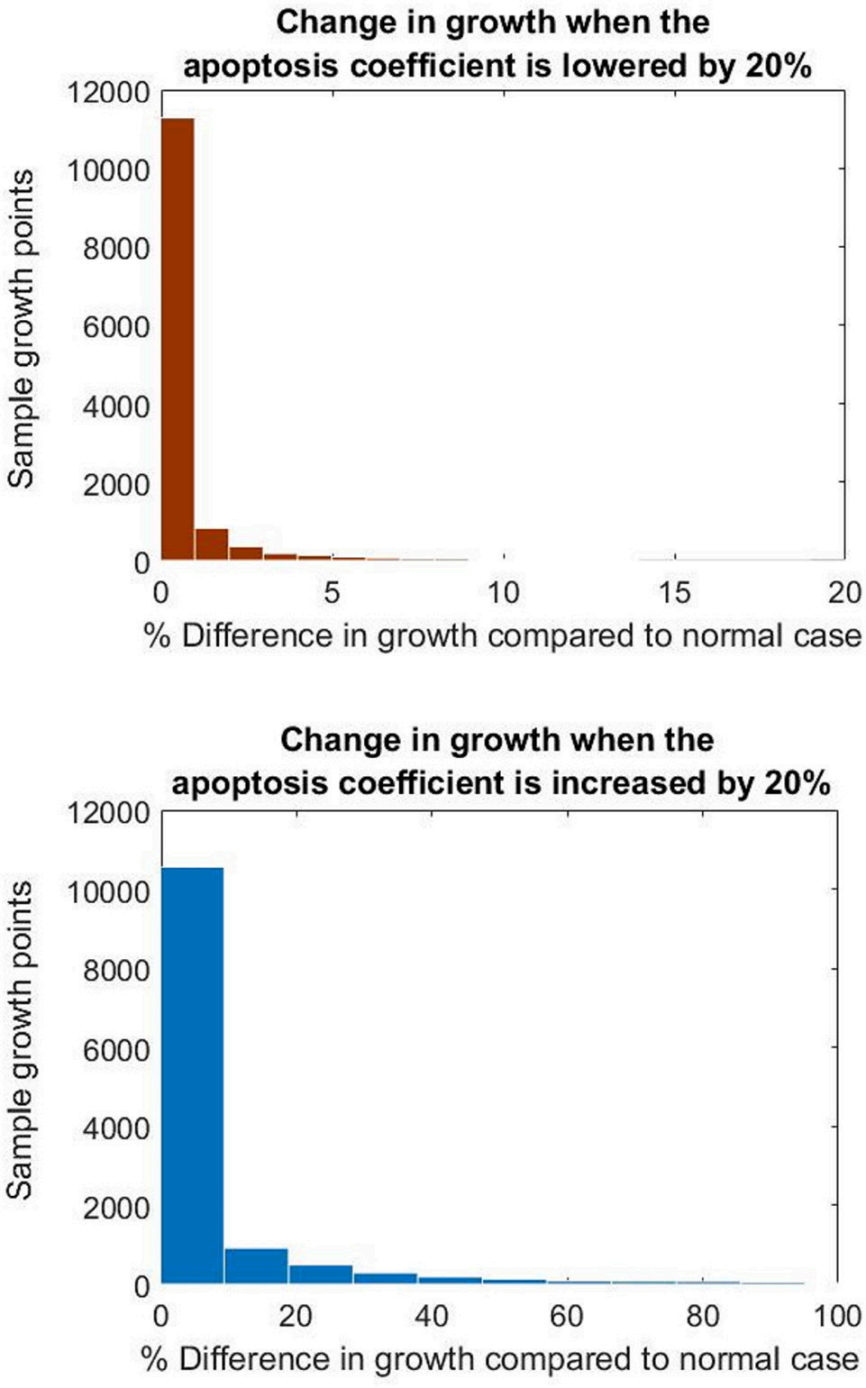
**Histograms showing the difference in growth at the nodes when changing the apoptosis parameter**.

As seen in Figure 12, NIH growth was sensitive to changes in both the apoptosis and proliferation coefficients. In particular the growth rate was more sensitive to the increase in the apoptosis parameter, with values increasing by up to three folds when the parameter was increased by 20%.

These results show how important is the influence of the biochemical parameters on the final clinical endpoint simulated by this model. So far, with these results it is not possible to conclude about how different smooth muscle cell behaviors could have a different impact on disease progression. However, if a more detailed biochemical model is proposed, the same kind of sensitivity analysis might enable us to draw conclusions about the influence of specific biological/physiological mechanisms (e.g., intra/extracellular mechanisms or processes related to inflammation) that play a key role in NIH.

The multi-scale modeling approach proposed here, has shown how using a mathematical description capturing different biological and physiological scales related to NIH can be used to have a better holistic understanding of the disease allowing the use of advanced computational tools to generate dynamic results related to disease progression. This kind of approach is not only useful for simulation (through validation with clinical data) but also for further analyses (e.g., sensitivity analyses) that can be linked to testing specific biological hypotheses.

### 4.1. Limitations

The voxel size of the CT images was 0.8 mm, which is very close to the dimensions of the arterial geometry analyzed. In addition, although CT scans were available of both pre and post-surgery, only post-surgery scans were used, and the geometry right after surgery was recreated as described in the method section. While this method to obtain the initial images is subject to inaccuracies due to both the remodeling occurring within the tissue and the quality of CT scans, the resolution of the images was the highest available for this study, as no other data acquisition techniques outside of those already part of the standard clinical procedure were used.

Results from the multi-scale approach using the biochemical model proposed in this paper are very encouraging even if only one type of cells was considered, i.e., smooth muscle cells. A next extension of this part of the model might consider additional types of cells contributing to NIH (e.g., fibroblasts, macrophages) (Model and Dardik, 2012).

An additional extension to this model could include the description of the endothelium and different functions related to this layer, for example the release of molecules that affect cell response and growth (Cui et al., 2014), the regulation of leukocyte adhesion after injury (Tseng et al., 2014), and the control of the uptake of immune cells, i.e., monocytes that later differentiate into macrophages in the vascular wall (Jaipersad et al., 2014).

As this is a first attempt to test the model, the time taken for simulations could be further improved. For instance, in the case of the left leg bypass the large number of mesh elements caused the process to be more computationally expensive compared to the case of the right leg bypass. In order to minimize computational time so that it would remain within values for potential clinical use, only one remodeling cycle was performed for the left side graft. Although this should not have affected the results, optimizing simulation time is key for improving the model.

The introduction of the impedance condition could also be added as part of the boundary conditions (Du et al., 2015). In addition, although the femoral artery is considered a large artery and a Newtonian model of blood flow should be sufficient, it could be interesting to compare simulations run using different non-Newtonian models against the current Newtonian model to evaluate whether there are any differences in the results. The use of non-Newtonian models has been shown to lead to different results in arterial flow studies. Newtonian models have already been shown to overestimate WSS in a previous study (Xiang et al., 2011), and the same might be happening in this case, especially at points where the diameter of the vessel becomes very small.

Another feature that might improve the model could be the introduction of moving walls, to mimic the pulsating movement of the arterial wall. Including the movement of solid boundaries in CFD simulations of arteries has been shown to improve results in previous models of cardiovascular disease (Alimohammadi et al., 2015), and the same is likely to be valid for NIH. It should be noted that the boundary conditions at the inlet also affect the outcome of the simulation. Limitations linked to the data acquisition and quality of ultrasound measurements themselves can be significant and there is additional uncertainty introduced due to the smoothing of the ultrasound data.

Finally, it is important to mention that the issue of patient-specific biomarkers is essential when trying to understand peripheral arterial disease and graft failure in individual patients. In these patients circulating biomarkers have been found to correlate with disease presence and severity (Owens et al., 2007). There is also evidence that certain circulating biomarkers may predict development of restenosis/graft disease; potential candidates include C-reactive protein, inflammatory cytokines, growth factors and adhesion molecules (Willigendael et al., 2005; Wildgruber et al., 2007). Further work will consist on collecting longitudinal datasets including imaging data and biomarkers in order to better represent and understand the biological aspects of the disease and to correlate them with hemodynamic variables.

## 5. Conclusion

The study presented a multi-scale model of the behavior of peripheral vein grafts affected by NIH when subject to WSS, the first to date to simulate the disease progression based on the dynamics of the flow in patient-specific geometries. A comparison intra-patient between a femoro-popliteal and femoro-distal graft was carried out which showed the potential but also, limitations of the applicability of the model to different cases. By means of this model it has been possible to analyze NIH progression and its mechanisms from a hemodynamics and a biochemical standpoint. In the future, the model will be used for the analysis of more patients in order to test its applicability on different cases of vein graft NIH. Moreover, the remodeling framework produced could be expanded in future work, for instance by extending the model to other components of vascular tissue. Mathematical models already exist that describe the cellular behavior in atherosclerosis (Cilla et al., 2013; Díaz-Zuccarini et al., 2014; Pichardo-Almarza et al., 2014). These could be modified to describe behavior of additional cells such as macrophages in NIH, in order to include a better description of the inflammation processes triggered in the disease. In addition, the endothelial layer could be added as already seen in previous literature (Pichardo-Almarza et al., 2014; Alimohammadi et al., 2016) to describe the transport of biochemical species into the vascular tissue. Modeling the endothelium would also enable to take into account the effect of WSS on the behavior of monocytes, as previously done for atherosclerosis (Díaz-Zuccarini et al., 2014), thus considering a further aspect of the mechanical response of cells. Further analysis could be done by considering other shear stress indices, as some of these have been shown to give better predictions in models of other vascular diseases such as atherosclerosis (Alimohammadi et al., 2016), which is also a disease affected by mechanical forces similarly to NIH, although through different cell species and processes. More flow characteristics could be included to describe recirculation and separation zones, all of which have been shown to affect the development of NIH (Murphy and Boyle, 2010). Finally, future work should investigate the use of fluid structure interaction in order to capture the movement of the arterial wall when subject to hemodynamic forces, which might have an influence in the results.

## Ethics statement

This study was carried out in accordance with the recommendations of the Institutional Human Investigation Committee (Veterans Affairs Connecticut Healthcare System, West Haven, CT, USA) with written informed consent from all subjects. All subjects gave written informed consent in accordance with the Declaration of Helsinki. The protocol was approved by the Institutional Human Investigation Committee (Veterans Affairs Connecticut Healthcare System, West Haven, CT, USA).

## Author contributions

FD, CP, and VD conceived the study and carried out simulations and wrote the manuscript. MB created the geometries from CT scans, AD acquired the clinical data and provided medical input. SH conceived the study and provided medical input.

## Funding

This project is supported by the Engineering and Physical Sciences Research Council (EPSRC) through the Doctoral Training Programme “Healthcare Engineering for an aging population.” The authors are grateful for the support of the EPSRC Network “POEMS” (EP/L001101/1) for facilitating the visit of AD to UCL. The authors gratefully acknowledge support by the EPSRC grant “Personalized Medicine Through Learning in the Model Space” (grant number EP/L000296/1) and the Leverhulme Trust Senior Research Fellowship “Exploring the Unknowable Using Simulation: Structural Uncertainty in Multiscale Models” (Fellowship number RF-2015-482). This work was supported by the resources and the use of facilities at the Veterans Affairs Connecticut Healthcare System (West Haven, CT).

### Conflict of interest statement

The authors declare that the research was conducted in the absence of any commercial or financial relationships that could be construed as a potential conflict of interest.
